# Bexarotene normalizes chemotherapy-induced myelin decompaction and reverses cognitive and sensorimotor deficits in mice

**DOI:** 10.1186/s40478-020-01061-x

**Published:** 2020-11-12

**Authors:** Angie C. A. Chiang, Alexandre V. Seua, Pooja Singhmar, Luis D. Arroyo, Rajasekaran Mahalingam, Jian Hu, Annemieke Kavelaars, Cobi J. Heijnen

**Affiliations:** 1 Division of Internal Medicine, Department of Symptom Research, University of Texas M.D. Anderson Cancer Center, 6355 MD Anderson Blvd, Unit 1055, Houston, TX 77030 USA; 2 Department of Cancer Biology, University of Texas M.D. Anderson Cancer Center, Houston, TX 77030 USA; 3 Programs of Cancer Biology and Neuroscience, MD Anderson Cancer Center UTHealth Graduate School of Biomedical Sciences (GSBS), Houston, TX 77030 USA

## Abstract

Frequently reported neurotoxic sequelae of cancer treatment include cognitive deficits and sensorimotor abnormalities that have long-lasting negative effects on the quality of life of an increasing number of cancer survivors. The underlying mechanisms are not fully understood and there is no effective treatment. We show here that cisplatin treatment of mice not only caused cognitive dysfunction but also impaired sensorimotor function. These functional deficits are associated with reduced myelin density and complexity in the cingulate and sensorimotor cortex. At the ultrastructural level, myelin abnormalities were characterized by decompaction. We used this model to examine the effect of bexarotene, an agonist of the RXR-family of nuclear receptors. Administration of only five daily doses of bexarotene after completion of cisplatin treatment was sufficient to normalize myelin density and fiber coherency and to restore myelin compaction in cingulate and sensorimotor cortex. Functionally, bexarotene normalized performance of cisplatin-treated mice in tests for cognitive and sensorimotor function. RNAseq analysis identified the TR/RXR pathway as one of the top canonical pathways activated by administration of bexarotene to cisplatin-treated mice. Bexarotene also activated neuregulin and netrin pathways that are implicated in myelin formation/maintenance, synaptic function and axonal guidance. In conclusion, short term treatment with bexarotene is sufficient to reverse the adverse effects of cisplatin on white matter structure, cognitive function, and sensorimotor performance. These encouraging findings warrant further studies into potential clinical translation and the underlying mechanisms of bexarotene for chemobrain.

**Electronic supplementary material:**

The online version of this article (10.1186/s40478-020-01061-x) contains supplementary material, which is available to authorized users.

## Introduction

During the last decade cancer treatment has become more and more successful, but unfortunately a large number of cancer survivors reports long lasting neurotoxic side effects of treatment, including cognitive impairment and sensory and motor abnormalities [28, 40, 41, 50, 51, 53, 54, 60]. There are no FDA-approved drugs to prevent or reverse these neurotoxicities. Therefore, development of novel therapeutic strategies is urgently needed.

We have recently shown that treatment of mice with cisplatin induces a profound and long lasting impairment in performance in tasks of spatial memory and executive functioning [5, 7, 8, 35, 65]. At the structural level, these behavioral deficits are accompanied by a decrease in dendritic spine density in the cingulate cortex, and a reduction in the expression of markers of synaptic integrity like PSD95 and synaptophysin in prefrontal cortex and hippocampus [5, 7, 35, 65]. In addition, we observed an increase in the coherency of fibers after staining for myelin basic protein in the cingulate cortex, indicating a reduction in arborization and complexity of myelinated axons [7].

Restoration of myelin damage as a result of cerebral insults or neurodegenerative processes is key to restoration of brain function. In the brain, myelin is produced by oligodendrocytes, while astrocytes, T cells and macrophages/microglia can all modulate myelin formation [15, 29, 45]. Retinoid X receptor (RXR) is a member of the NR2B nuclear receptor family. As a common binding partner of many other nuclear receptors, it mainly functions as a ligand-dependent transcription factor and regulates many physiological processes. Activation of the RXR family of receptors can promote (re)myelination either via their anti-inflammatory effect, their effects on monocyte/macrophage phagocytosis of myelin to remove myelin debris, and for their capacity to directly stimulate oligodendrocyte precursor proliferation/differentiation [9, 12, 39]. RXR activation, either via genetic manipulation or pharmacologic interventions, increased oligodendrocyte differentiation and remyelination in models of toxin-induced demyelination in rats [25]. Moreover, transcripts encoding RXRγ were upregulated during remyelination and expressed by cells of the oligodendrocyte lineage [25].

Bexarotene is a synthetic retinoid modulator of RXRs that binds the RXR receptor subtypes RXRα, RXRβ, and RXRγ with high affinity. The drug has been explored as an add on for cancer therapies. Although some studies indicated stabilization of advanced small cell lung carcinoma in patients receiving add on bexarotene, there was no detectable increase in survival. Importantly, there is no evidence that bexarotene negatively interferes with anti-cancer effects of chemotherapy [16, 36, 43]. RXR are expressed in all cell types in the brain, including neurons, oligodendrocytes, astrocytes and microglia (http://dropviz.org/). In vitro, RXR stimulation by the agonist bexarotene restored the age-related deficiencies in myelin debris phagocytosis by macrophages, a key process in myelin maintenance [38]. A more recent study showed that bexarotene also promotes myelin formation in a genetic model of myelin loss [66]. Moreover, RXR agonists like bexarotene can reduce the cognitive deficits and brain damage that develop in rodent models of cerebral ischemia, subarachnoid hemorrhage, and traumatic brain injury [9, 58, 63, 67].

The aim of this study was to better characterize the white matter damage that develops in mice treated with cisplatin and to determine whether the RXR agonist bexarotene reverses these white matter alterations. As a functional readout we analyzed not only cognitive function but also sensorimotor function in cisplatin-treated mice. Potential white matter damage including changes in myelin structure were investigated in the sensorimotor cortex as well in view of the abnormalities in sensorimotor function reported by patients treated with chemotherapy. Bexarotene is already FDA-approved as a treatment for cutaneous T cell lymphoma and is not likely to interfere with the efficacy of cancer treatment [16, 17]. Therefore, bexarotene could represent a promising new and safe therapeutic strategy to reverse the negative consequences of cancer treatment for brain health without negative interference with cancer treatment.

## Materials and methods

### Mice

Male and female C57BL/6 J mice (Jackson Laboratory) were housed at 22 ± 2 °C on a 12/12 h reverse dark–light cycle with water and food ad libitum. All experiments were conducted at The University of Texas MD Anderson Cancer Center and approved by the Institutional Animal Care and Use Committee of The University of Texas MD Anderson Cancer Center in Houston, TX. Mice were randomly assigned to treatment groups and investigators were blinded to treatment.

### Chemotherapy and bexarotene treatment

At 9 weeks of age, mice received cisplatin (Fresenius Kabi USA) or phosphate-buffered saline (PBS) administered intraperitoneally (i.p.) in 2 rounds consisting of 5 daily doses of 2.3 mg/kg, followed by 5 days of rest without injections. The cumulative dose of 23 mg/kg is equivalent to 70 mg/m^2^ in humans [37], which is within the range of one cycle of cisplatin treatment in humans [55]. We showed previously that this treatment regimen has antitumor effects in the mouse and induces cognitive deficits [7, 8, 35].

Bexarotene in 10% DMSO in sunflower seed oil was delivered at a final dose of 100 mg/kg/day for 5 consecutive days by oral gavage starting 24 h after the last dose of cisplatin.

### Tissue processing and Black Gold II staining

Mice were sacrificed after behavioral testing using brief CO2 exposure, followed by intracardial perfusion with ice-cold PBS. Brains were removed and post-fixed in 4% PFA for 48 h, cryoprotected in sucrose, and cut at 25 μm in the coronal plane on a sliding microtome. For each animal, 4 sections were used for Black Gold II (Millipore, #AG105) staining according to manufacturer’s instructions. Briefly, sections were mounted onto slides and dried overnight at room temperature. The next day, slides were rehydrated in ddH2O before immersion in Black Gold II solution at 60 °C for 15 min. After washing in ddH2O, slides were incubated in pre-warmed 1% sodium thiosulfate solution at 60 °C for 3 min. Slides were then rinsed with ddH2O and dehydrated through a series of ethanol and xylene and coverslipped with Permount. Bright field images were taken using EVOS^®^ FL Auto microscope and percent area and coherency were quantified using ImageJ with the OrientationJ plugin.

### Transmission electron microscopy

For TEM analysis of myelin integrity, mice were anesthetized and transcardially perfused with PBS. One hemisphere of the brain was post-fixed in 2% glutaraldehyde plus 2% PFA in PBS at 4 °C for at least a week. Small biopsy sample extracts about 1 mm in diameter and 2 mm in length were dissected out from the motor cortex. Fixed samples were processed at the High Resolution Electron Microscopy Facility at MD Anderson. Briefly, samples were washed in 0.1 M sodium cacodylate buffer and treated with cacodylate buffered tannic acid, post-fixed with 1% buffered osmium and stained en bloc with 0.1% Millipore-filtered uranyl acetate. Samples were then dehydrated in increasing concentrations of ethanol and infiltrated and embedded in LX-112 medium. Samples were polymerized in a 60 °C oven for approximately 3 days. Ultrathin sections were cut using a Leica Ultracut microtome and then stained with uranyl acetate and lead citrate in a Leica EM Stainer. Stained samples were examined in a JEM 1010 transmission electron microscope (JEOL USA, Inc, Peabody, MA) using an accelerating voltage of 80 kV. Digital imaged were obtained using an AMT imaging system (Advanced Microscopy Techniques Corp., Danvers, MA). Percentage of axons with damaged myelin sheaths and myelin sheath thickness (µm) were determined using image analysis software (Image J). Percent damaged myelin was quantified as (number of axons with decompacted/loosened myelin/total number of myelinated axons) * 100. For myelin thickness, maximum myelin diameter was used to measure thickness. The g ratio was quantified as the ratio of axonal/axonal + myelin diameter. We scored on average 18 axons from 3 to 4 images per animal.

### Lipidomics

Dissected forebrains were snap frozen in liquid nitrogen and stored at − 80 °C. In order to identify and quantify a wide range of lipids, brain extracts were prepared and analyzed by liquid chromatography coupled with high-resolution mass spectrometry (LC-HRMS) for a full scale lipidomics profiling. Approximately 20 mgs of tissue sample was homogenized with Precellys Tissue Homogenizer. Lipids were extracted using a mix of ice cold MtBE (Methyl tert-butyl ethe)/Methanol/Water. Samples were centrifuged at 17,000 g for 5 min at 4 °C, and the organic top layer was transferred to a clean tube, followed by evaporation to dryness under nitrogen. Samples were then reconstituted using isopropanol, and 5 µL was injected into a Thermo Vanquish liquid chromatography (LC) system containing an Accucore C30 2.1 × 150 mm column with 2.6 µm particle size. Mobile phase A was 60/40 Acetonitrile/water and mobile phase B was 90/10 Isopropanol/Acetonitrile. Both mobile phases A and B contained 10 mM Ammonium formate and 0.1% formic acid. The flow rate was 200 µL/min (at 35 °C), and the gradient conditions were from 40% MPB to 100% MPB in 50 min and hold at 100% B for 10 min. The total run time was 70 min. Data was acquired using a thermo Orbitrap Fusion Tribrid mass spectrometer under ESI positive and negative ionization mode at a resolution of 240,000. Raw data files were imported into Thermo Lipid Search software for lipid analyses.

### RNA sequencing and data analysis

PFC samples were collected 1 h after the last dose of bexarotene (Day 5) for RNA sequencing. Briefly, mice were euthanized with CO2 exposure, followed by intracardial perfusion with ice-cold PBS. Brains were removed, and hemi-cortices were micro-dissected on an ice-cold metal plate and pre-frontal cortex was immediately placed in RNA later (Qiagen). Tissue was homogenized in TRIzol reagent (Invitrogen) and total RNA was extracted using the RNeasy MinElute Cleanup Kit (Qiagen). A 72 bp, paired-end, stranded cDNA library was prepared from extracted RNA using the Stranded mRNA-Seq kit (Kapa Biosystems, Wilmington, MA) and sequenced on Illumina HiSeq 4000.

### Behavior

#### Beam walking

On training day 1, each mouse is trained to cross a beam consisting of 85 cm in length with a flat surface of 1.2 cm in width that rests above the table-top on two poles. Training repeats for 3 times. Mice are placed at one end of a beam and the time required to cross to the escape platform on the other end is measured. Mice are then trained on a thinner beam 0.6 cm in width before progressing to a more difficult round beam 0.6 cm in diameter. On test day, the test beam is replaced by a more difficult round rod measuring 85 cm × 0.4 cm. The time to cross the rod is recorded and three trials are averaged. The beams are cleaned with 70% ethanol before the next animal is tested.

#### Puzzle box test

The puzzle box test was used to measure executive functioning as we described before [7]. Mice are placed into a brightly lit arena (55 cm × 28 cm) connected to a small dark area (15 cm × 28 cm) by an underpass (4 cm × 2.5 cm). In the easy trials (trials 1–4), the underpass is open and freely accessible. During intermediate trials (trials 5–7), the underpass is filled with bedding, requiring mice to burrow through to enter the dark compartment. In the difficult trials (trials 8–11), the underpass is closed and covered by a lid that the mice need to unplug and remove before they can enter the tunnel. The time elapsed before the animal enters the dark compartment is recorded as a measure of executive function.

#### Novel object and place recognition task (NOPRT)

The NOPRT to assess spatial and working memory was performed as described [7, 35]. During training, mice were placed in the testing arena for 5 min with two identical objects placed on the same side of the arena. Mice were returned to their home cage for 30 min and tested in the arena with one familiar object placed at the same location as before, and one novel object placed on the opposite end of the arena. The investigation time (T) toward either object during the 5-min testing phase was evaluated using EthoVision XT 10.1 video tracking software (Noldus Information Technology Inc., Leesburg, VA). Discrimination index was determined as (T_Novel_ − T_Familiar_)/(T_Novel_ + T_Familiar_).

### Statistical analysis

Statistical analyses for behavioral and myelin data were performed using two-way ANOVA followed by Tukey test or using Mann–Whitney U test where appropriate in GraphPad Prism 7.01. RNA sequencing data analysis was performed using raw reads on the FASTQ format. The quality of the reads was evaluated with FastQC [1]. The sequencing reads were mapped to the mouse reference genome (mm10 version) using STAR package [14]. The uniquely mapped reads were retained and the featureCounts program from the Subread package [31] was used for counting mapped reads. The gene count normalization and differential gene expression calculation of PBS versus CIS and CIS versus BexCIS comparisons were performed using DESeq 2 package [33]. We excluded genes with read counts less than 10 and selected genes with adjusted *p* value < 0.1 as differentially expressed. The pathway enrichment analysis was performed using Ingenuity Pathway Analysis tool (IPA; Qiagen Inc.).

## Results

### Cisplatin reduces myelin density and compaction

**Fig. 1:**
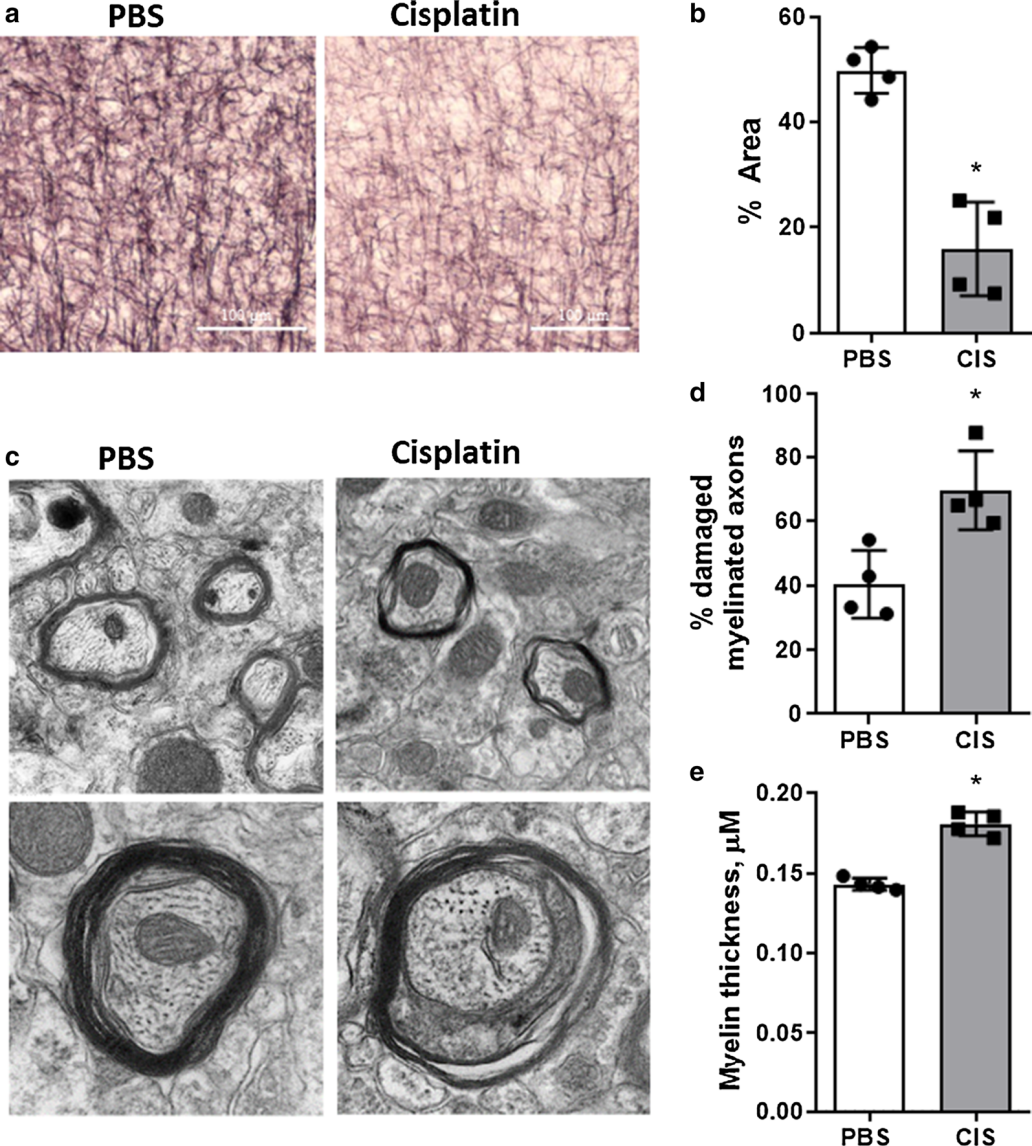
Cisplatin induced white matter changes in myelin density and sheath ultrastructure. **a** Representative images of Black Gold II staining for myelin in the sensorimotor cortex of mice (n = 4) treated with either two 5-day cycles of PBS or cisplatin. Scale bar = 100 μM. **b** Percent area positive for Black Gold II was measured in the sensorimotor cortex. **c** Representative transmission electron microscopy (TEM) images were used to analyze and compare the ultrastructure of myelinated axons in the cortex of PBS and cisplatin treated animals. **d** Percent of damaged myelinated axons and **f** myelin thickness were quantified to illustrate the differences between the two treatment groups. MannWhitney U test: *p < 0.05. Results expressed as individual data points and mean ± SD

We previously showed that cisplatin treatment reduces integrity of lipid structures in the cingulate cortex visualized by staining with the lipophilic dye Black Gold II that has high affinity for myelin [7, 49]. The results in Fig. 1 show that cisplatin also induces abnormalities in Black Gold II staining in the prefrontal cortex. We next examined the impact of cisplatin treatment on ultrastructural changes in myelin within the prefrontal cortex by transmission electron microscopy (TEM). Interestingly, we did not detect hypomyelinated layers in the prefrontal cortices of cisplatin-treated mice. Instead, we observed ultrastructural myelin abnormalities characterized by an increased percentage of axons with split sheathes and myelin decompaction in cisplatin-treated mice (Fig. 1a, b). Myelin lamellae were broken at the innermost region of the myelin sheath of cisplatin treated animals and myelin wrappings exhibited a protracted phenotype. Quantification of the thickness of the myelin showed a significant increase in myelin thickness in cisplatin-treated as compared to PBS-treated mice due to the loosening of myelin sheath layers (Fig. 1c).

### Cisplatin-induced changes in gene expression in the prefrontal cortex

RNAseq analysis of the transcriptome in the prefrontal cortex of cisplatin and PBS-treated mice identified differential expression of only 27 genes (adjusted *p* < 0.1; Table 1; Additional file 1: Supplementary Figure 1). The top upregulated gene is *Cdh1*, the gene encoding E-cadherin. In myelinating Schwann cells, E-cadherin is a component of adherens junctions that stabilizes the architecture of non-compact myelin regions [3]. *Mal* is another differentially expressed gene; it encodes MAL, a protein produced by oligodendrocytes that is involved in myelin compaction [47]. Expression of *Ptgds*, the gene encoding prostaglandin D2 synthase was upregulated in the cisplatin-treated group and this enzyme functions as a trophic factor in the central nervous system. It is known to be involved in peripheral nervous system myelination [4, 56]. The topmost downregulated gene in our RNA seq analysis is *Lcn2*, which encodes lipocalin 2 (Table 1). Lipocalin 2 is a selective modulator of activation of peroxisome proliferator-activated receptor-γ, a member of the family of RXR receptors and functions in lipid homeostasis and energy expenditure [27].

**Table 1:** Differentially expressed genes comparing prefrontal cortex in response to cisplatin

Gene	log2FoldChange	Padj
Upregulated genes		
*Cdh1*	1.281674	9.94E−05
Hba-a2	1.233547	0.05081
Slc47a1	1.145545	0.004625
Gm42047	0.780093	0.027633
Prg4	0.757131	0.009389
Fmod	0.528089	1.30E−06
Rsph10b	0.457828	0.098236
Islr	0.423915	0.079967
Slc13a4	0.421721	0.086691
Hsd11b1	0.372621	0.086691
*Ptgds*	0.354773	0.001266
Slc26a2	0.310505	0.068721
Uvssa	0.280211	0.098236
Cxcl12	0.237664	0.086691
Downregulated genes		
Lcn2	− 3.75254	0.003447
Capn11	− 3.04156	0.007803
Ifitm1	− 0.83312	0.082431
Hspa1a	− 0.70256	0.003447
mt-Ti	− 0.51871	0.027633
Xdh	− 0.44559	0.060968
Dusp1	− 0.38506	0.086691
Dusp5	− 0.3716	0.086691
Tbc1d4	− 0.30415	0.019153
*Mal*	− 0.28939	0.079967
Fam107a	− 0.28644	0.086691
Bc1	− 0.26192	0.009824
Nr1d1	− 0.2582	0.009796

Mice were treated with cisplatin (two rounds of 5 daily injections of 2.3 mg/kg with 5 days of rest in between) or PBS and prefrontal cortex was collected 5 days after the last dose of cisplatin. Data represent genes differentially expressed genes with padj < 0.1. Genes identified in italic are involved in myelin formation/maintenance

In line with what can be expected for small gene sets, IPA analysis of the total set of 27 differentially expressed genes did not identify significantly enriched pathways.

### Cisplatin-induced myelin loss and decompaction is normalized with bexarotene treatment

**Fig. 2:**
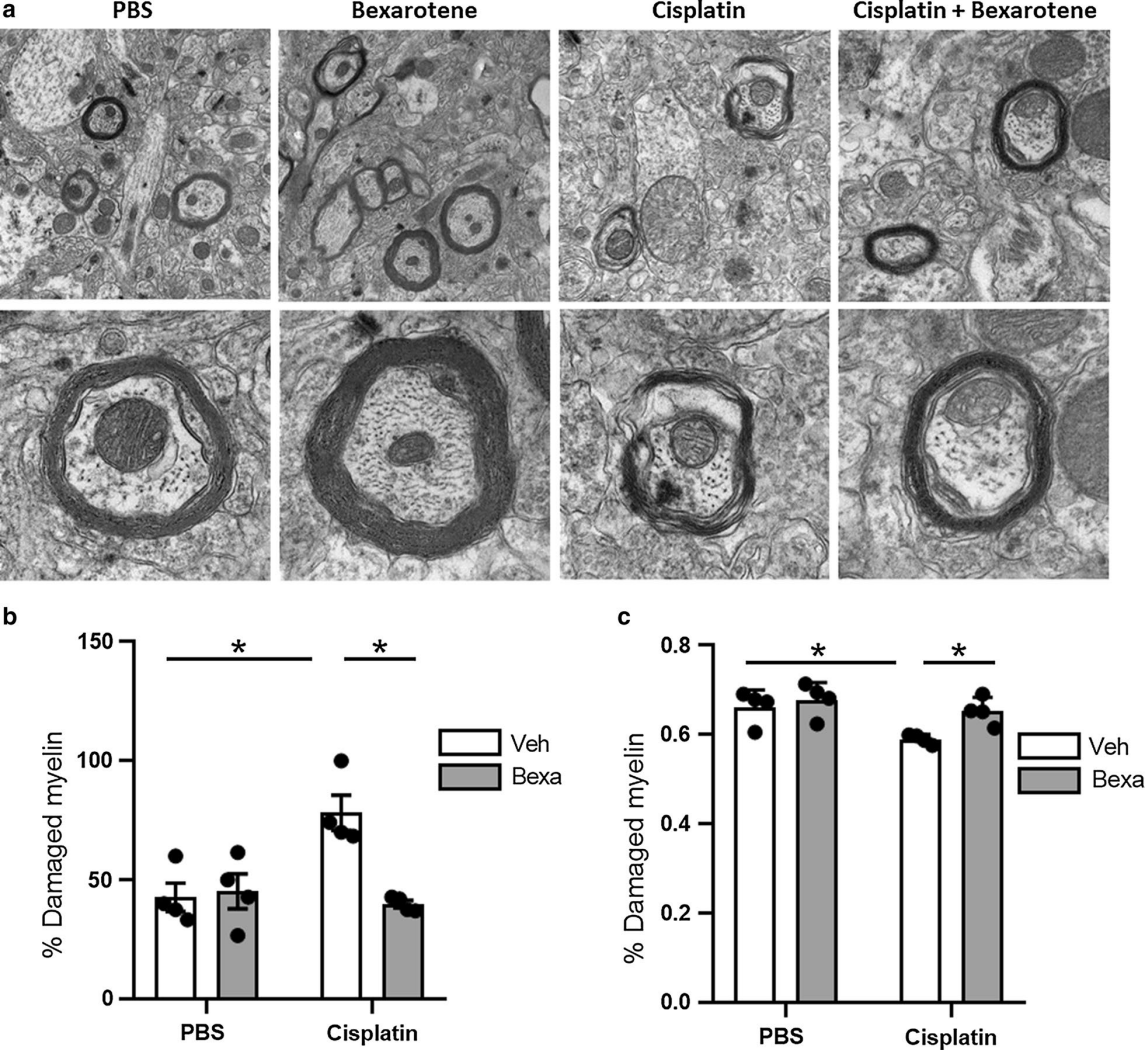
Reversal of myelin decompaction in response to bexarotene administration to cisplatin-treated mice. Representative TEM images (**a**), quantification of the percentage of damaged myelinated axons (**b**) and g ratio of myelinated axons in the prefrontal cortex following cisplatin and bexarotene treatment. Percent of damaged axons was quantified, and results are represented as mean ± SD; n = 4. Two-way ANOVA followed by Tukey’s multiple comparisons test. *p < 0.05, **p < 0.01

Next we determined the effect of the RXR agonist bexarotene on the cisplatin-induced abnormalities in myelin in the sensorimotor cortex. Mice were treated with two cycles of cisplatin followed by 5 days of treatment with bexarotene (100 mg/kg/day i.p.) starting 24 h after the last dose of cisplatin. In line with the data in Fig. 1, TEM analysis of the ultrastructure of myelin sheaths of this independent group of mice revealed that cisplatin treatment results in damaged myelinated axons with split sheaths and myelin decompaction and a reduction in the g ratio (Fig. 2). Notably, 5 days of bexarotene administration normalized all aspects of the cisplatin-induced abnormalities in myelin ultrastructure (Fig. 2).

**Fig. 3:**
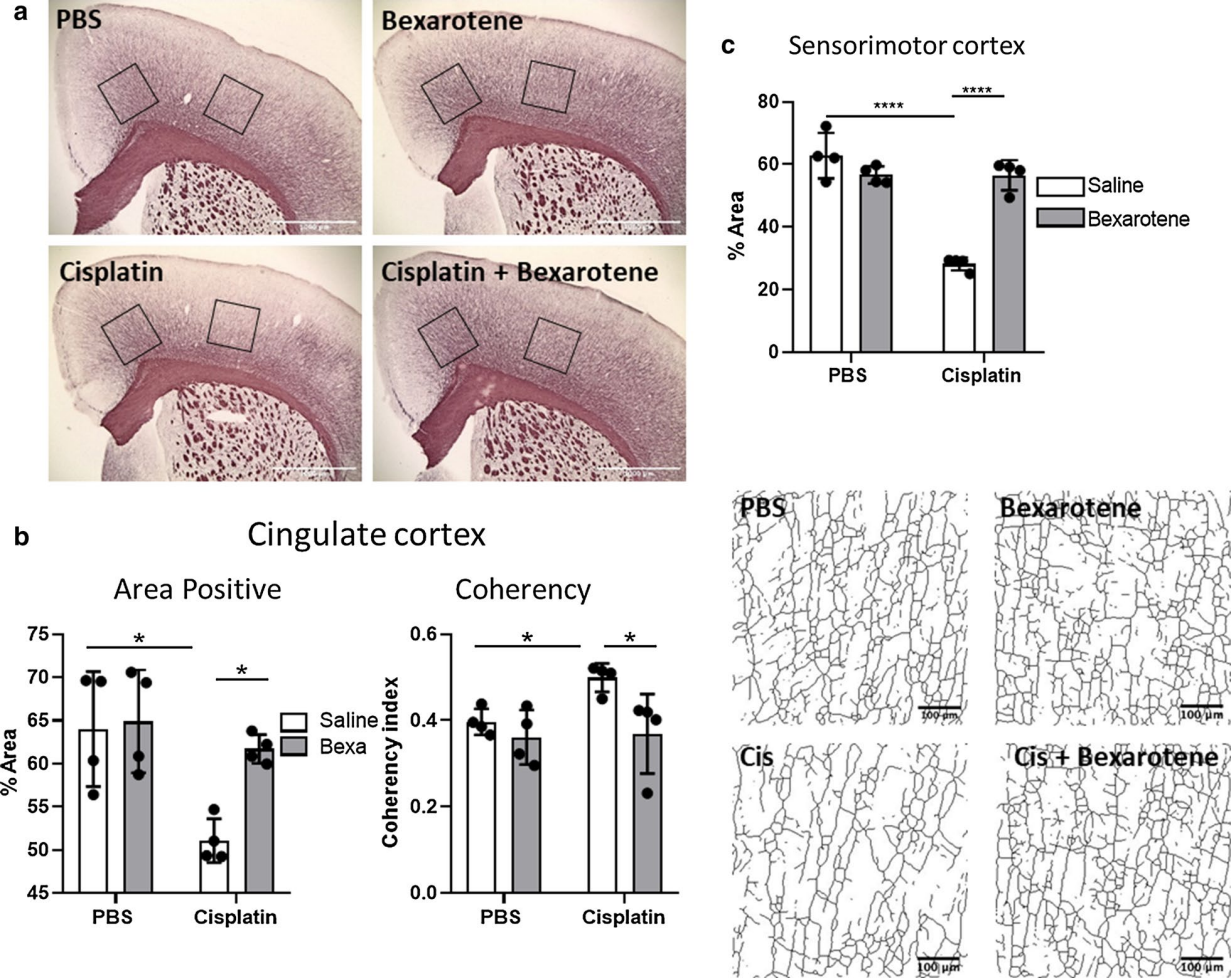
Bexarotene reverses white matter damaged induced by cisplatin treatment. **a** Representative overview images of myelin staining using Black Gold II following bexarotene treatment in cisplatin-treated animals (n = 4). Black squares indicate the two areas analyzed—cingulate and sensorimotor cortices. Scale bar = 1000 μM. **b**, **c** Quantification of percent positive area to indicate myelin density in the cingulate (**b**) and sensorimotor (**c**) cortex. For the cingulate cortex, images were skeletonized and the coherency index was quantified to assess the complexity of fibers (Scale bar = 100 μM). Results are expressed as mean ± SD. Two-way ANOVA followed by Tukey’s multiple comparisons test. *p < 0.05, ***p < 0.001, ****p < 0.0001

At the microscopic level, cisplatin treatment reduces the intensity of Black Gold II staining in the cingulate cortex ([7] and Fig. 3a, b) and has an even more pronounced effect on Black Gold II staining in the sensorimotor cortex (Fig. 3c). Five daily injections of bexarotene after completion of cisplatin treatment were sufficient to normalize the intensity of Black Gold II staining in the motor cortex (Fig. 3c) and in the cingulate cortex (Fig. 3a, b). Bexarotene treatment also normalized the cisplatin-induced increase in fiber coherency in the cingulate cortex (Fig. 3b).

### Bexarotene normalizes cognitive and sensorimotor function in mice treated with cisplatin

**Fig. 4:**
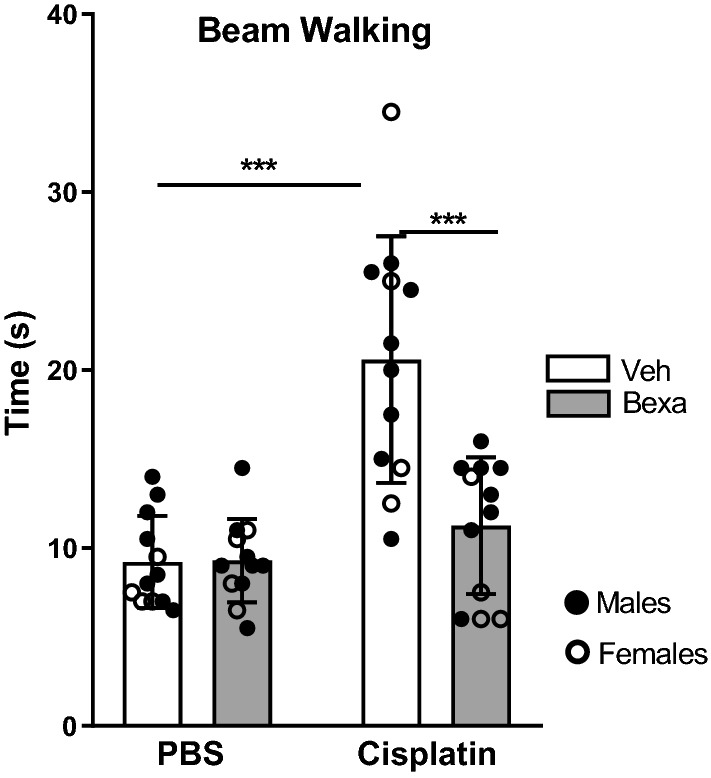
Bexarotene treatment reverses sensorimotor deficits induced by cisplatin. Mice were treated with cisplatin followed by bexarotene and performance in the beam walking test was assessed as a measure of sensorimotor function. Time to cross the beam was recorded (n = 8 males and 4 females). Performance on narrow beam walking test measured by time required to cross the beam. Results are represented as mean ± SD. Two-way ANOVA followed by Tukey’s multiple comparisons test. ***p < 0.001

To assess the effect of cisplatin and bexarotene treatment on fine motor coordination and balance, we used the beam walking test in which we recorded the time to traverse an elevated narrow round beam to an escape platform. The time needed to traverse the beam was significantly increased in cisplatin-treated mice (Fig. 4). Treatment with bexarotene normalized performance of cisplatin-treated male and female mice in the beam walking test (Fig. 4).

**Fig. 5:**
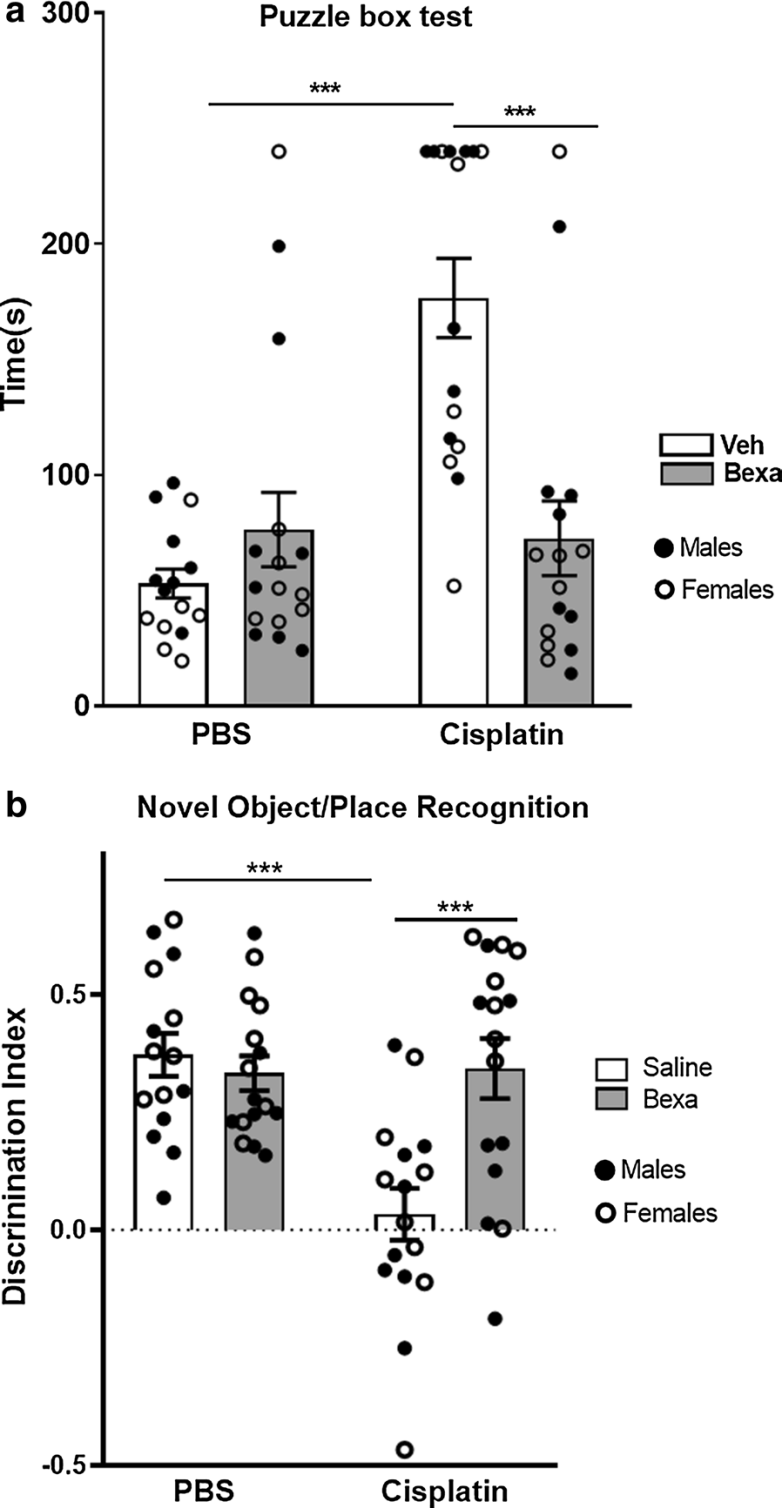
Bexarotene effects on cisplatin-induced cognitive impairments. Mice (n = 8 males and 8 females) were treated with cisplatin followed by bexarotene and performance in the puzzle box test for executive function and the novel object and space recognition test of short term memory and spatial orientation was assessed. **a** The puzzle box test started 7 days after the last dose of Bexarotene treatment. Entry time into the dark compartment in the easy and intermediate trials was similar between groups (See Additional file 1: Supplemental Figure 1). Time required for entry in two trials of the difficult task was recorded and the average time was calculated for each mouse. **b** NOPRT performance was tested in the week after completion of puzzle box test. The discrimination index was calculated as (T_Novel_ − T_Familiar_)/(T_Novel_ + T_Familiar_), with the value 0 indicating no preference for the novel object. Results are expressed as mean ± SEM. Two-way ANOVA followed by a Tukey’s multiple comparisons test. ***p < 0.001

In line with our previous studies, treatment with cisplatin significantly reduced performance in the puzzle box test used to assess executive function; the results in Fig. 5a demonstrates that during the difficult test, cisplatin-treated male and female mice required more time than control mice to enter the dark compartment. Performance of male and female mice treated with cisplatin followed by bexarotene was similar to that of control mice (Fig. 5a and Additional file 1: Supplementary Figure 2), indicating that bexarotene treatment normalized executive function in both sexes.

We used the novel object and place recognition test (NOPRT) to assess short term memory and spatial orientation. Cisplatin reduced the preference of male and female mice for the novel object. Treatment with bexarotene completely reversed the effect of cisplatin on performance in the NOPRT (Fig. 5b).

### Cisplatin and bexarotene do not induce major changes in lipid composition of the prefrontal cortex

**Fig. 6:**
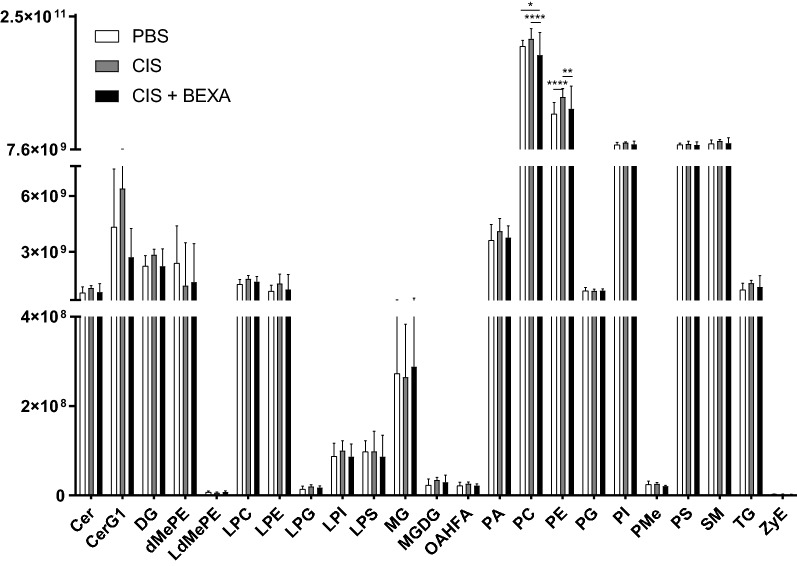
Lipidomic analysis of the PFC of mice treated with cisplatin and bexarotene. Quantification of the peak intensity of different lipid subclasses in prefrontal cortex of mice treated with cisplatin and bexarotene. Results are expressed as mean ± SD. n = 4 mice/group. *p < 0.05, **p < 0.01. *Cer* ceramides, *CerG1* monoglycosylceramide, *DG* diacyl-glycerols, *dMePE* dimethylphosphatidylethanolamine, *LdMePE* lysodimethylphosphatidyl-ethanolamine, *LPC* lysophosphatidylcholine, *LPE* lysophosphatidylethanolamine, *LPG* lysylphosphatidylglycerol, *LPI* lysophosphatidylinositol, *LPS* lipopoly-saccharide, *MG* monoradylglycerolipids, *MGDG* monogalactosyl diacylglycerol, *OAHFA* (O-acyl)-ω-hydroxy FA, *PA* phosphatidic acid, *PC* phosphatidylcholines, *PE* phosphatidyl-ethanolamines, *PG* phosphatidylgylcerols, *PL* phospholipids, *PMe* phosphatidyl-(N)-methylethanolamine, *PS* phosphatidylserine, *SM* sphingomyelin, *TG* triglyceride, *ZyE* zymosterol

Bexarotene has been shown to restore myelination and increase lipid levels in the spinal cord of mice with hypomyelination due to a genetic defect [66]. To determine whether the cisplatin-induced abnormalities in myelin structure and the restorative effects of bexarotene are also associated with changes in lipid composition, we performed lipidomic analysis of the prefrontal cortex. Samples were collected 1 h after the 5th dose of bexarotene. At this time point, there was no evidence for major decreases in lipid levels or major changes in lipid composition in the brain of cisplatin-treated mice as compared to PBS-treated mice. Moreover, administration of bexarotene to cisplatin-treated mice did not have major effects on lipids either. We only detected a very modest but statistically significant increase in 2 lipid species in the brain of cisplatin-treated mice The level of these two lipids was reduced by administration of bexarotene to cisplatin-treated mice (Fig. 6).

### Effect of bexarotene on transcriptome in PFC of cisplatin-treated mice

To get more insight in the pathways activated by bexarotene in cisplatin-treated mice, we performed RNA seq analysis on the fifth day of bexarotene treatment followed by IPA analysis to determine pathways, functional enrichment, and upstream regulators enriched in samples from mice treated with cisplatin followed by bexarotene- as compared to mice treated with cisplatin and vehicle. Bexarotene treatment induced differential expression of 713 genes (172 down and 541 up) (adjusted *p* < 0.1; Additional file 2: Supplementary Table 1). Five of these genes were also differentially expressed when comparing samples from mice treated with cisplatin alone versus PBS alone (Lcn2, Hspa1a, Dusp5, TBC1D4, and Cxcl12). Bexarotene reversed the effect of cisplatin for all five of these genes. Lcn2 was most strongly upregulated in response to bexarotene with a log2fold change of 3.29.

**Fig. 7:**
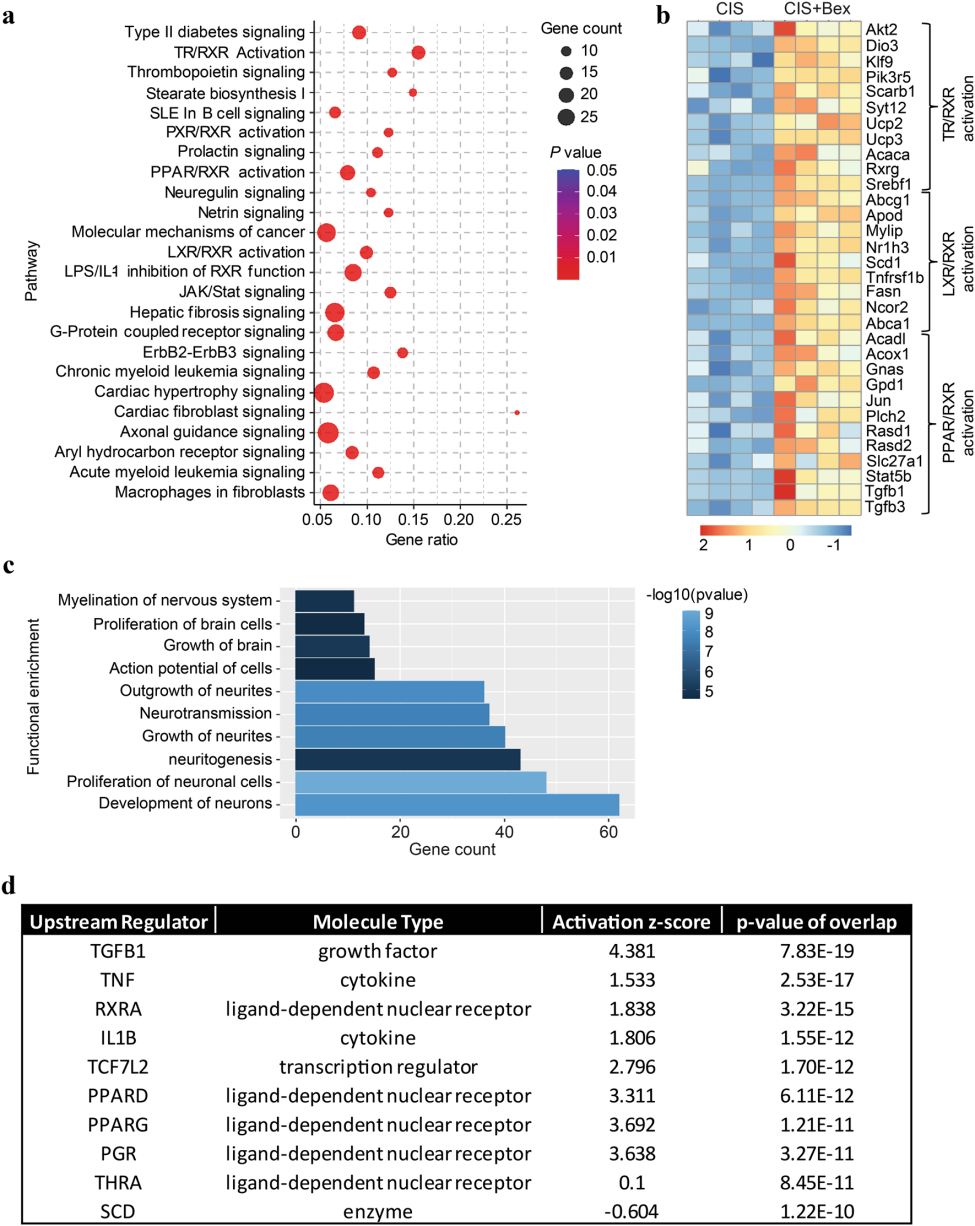
RNAseq analysis of the effect of bexarotene on the PFC transcriptome in cisplatin treated animals. Mice were treated with cisplatin and bexarotene and transcriptome analysis of the PFC was performed after the 5th dose of bexarotene and IPA pathway analysis was performed on the upregulated genes (padj < 0.1). **a** The dot plot shows the top up-regulated pathways identified in PFC from mice treated with cisplatin + bexarotene as compared to cisplatin alone using the IPA tool. The size of the dot represents gene count and the color represents the *p* value. **b** The heat map shows the expression profile of the differentially expressed genes related to the TR/RXR, LXR/RXR, and PPAR/RXR pathways from the comparison of PFC from mice treated with cisplatin versus cisplatin + bexarotene. **c** The bar diagram shows the functional enrichment related to the nervous system and development function identified in PFC from mice treated with cisplatin + bexarotene using the IPA tool. **d** Top upstream regulators predicted by the IPA tool to be causing bexarotene-induced gene expression changes

Consistent with bexarotene being an RXR agonist, pathway characterization of the 541 genes that were up-regulated in response to administration of bexarotene to cisplatin-treated mice identified activation of an RXR-heterodimer pathway, the TR/RXR-pathway, as one of the top canonical pathways affected (Fig. 7a). A heat map showing the relative expression of the genes in the TR/RXR, LXR/RXR and PPAR/RXR pathways is presented in Fig. 7b.

Pathway enrichment analysis of the upregulated genes also showed the activation of the Neuregulin Signaling pathway, which is implicated in myelination and synaptic function (Fig. 7a, Additional file 1: Supplementary Figure 3A). The Netrin pathway that is involved in providing axonal guidance cues and myelin maintenance was also predicted to be activated in mice treated with cisplatin and bexarotene (Fig. 7a, Additional file 1: Supplementary Figure 3B). Other pathways affected were G-Protein Coupled Receptor Signaling, and Axonal Guidance Signaling (Fig. 7a).

Functional enrichment analysis of the up-regulated genes showed the development of neurons, neuritogenesis, and myelination of nervous system (Fig. 7c) under the category of nervous system development and function in the IPA. Regulator analysis showed five out of top 10 upstream regulators driving transcriptional changes in cisplatin + bexarotene treated mice were related to the RXR network, namely, RXRa, THRA, PGR, PPARA, PPARG (Fig. 7d). PPAR forms heterodimers with RXR and acts as a regulator of fatty acid metabolism and overall energy homeostasis [26, 62].

## Discussion

An increasing number of cancer survivors suffers from persistent neurological impairments that include cognitive and sensorimotor deficits that reduce quality of life of cancer survivors. Therefore, there is a growing need for interventions that can mitigate these adverse effects of chemotherapy. Using a mouse model of cisplatin-induced cognitive impairment that we recently developed [8], we show here that cisplatin treatment also induces impairments in sensorimotor function. These cisplatin-induced functional abnormalities are associated with a decrease in white matter density in the cingulate and sensorimotor cortex. At the ultrastructural level cisplatin-treated mice show changes in the myelin structure represented as loosely wrapped irregular multilamellar myelin membranes around the axon.

In search for a way to reverse these cisplatin-induced deficits, we examined the effect of the RXR agonist bexarotene. Our data show that administration of only 5 daily doses of bexarotene after completion of cisplatin treatment is sufficient to normalize cognitive function, sensorimotor performance and myelin (ultra)structure. RNAseq analysis of the effect of bexarotene administration to cisplatin-treated mice on the transcriptome in the prefrontal cortex confirmed activation of RXR pathways. In addition, this transcriptome analysis showed that bexarotene activated pathways involved in myelination, axon guidance and synaptic function. Bexarotene is already approved for the treatment of cutaneous T cell lymphoma [17] and increased survival of a subgroup of patients treated with a platinum-based drug, indicating there is no negative effect on cancer treatment [43]. Therefore, rapid clinical translation of these findings should be possible.

We and others reported earlier that treatment of mice with cisplatin results in changes in myelin as assessed at the light microscopic level. Moreover, there is accumulating evidence for white matter abnormalities in patients treated for cancer including platinum-based chemotherapeutics [10, 30, 51]. To the best of our knowledge, this study is the first to describe that chemotherapy induces decompaction of myelin in the brain as detected at the electronmicrosopical level. Functionally, ultrastrucutral changes in myelin have been shown to affect conduction velocity and axonal protection. For example, the decompaction of myelin around the optic nerve of PLP-deficient mice was associated with a reduction of the neuronal conduction velocity [22]. Ultrastructural alterations of myelin, including interlaminar splitting of myelin sheaths indicative of myelin decompaction similar to what we observed here were reported in a mouse model of alcohol abuse [46]. Moreover, in white matter of the brains of human alcoholics, myelin membranes were also shown to be irregularly folded and split, indicating vacuoles between the myelin lamellae in association with enlarged mitochondria [44, 52]. Patients with a mutation of the neurofibromin gene (Nf1) in mature oligodendrocytes show white matter defects including myelin decompaction in conjunction with cognitive deficits and behavorial abnormalities [32].

In search for potential mechanisms of cisplatin-induced myelin decompaction, we compared the transcriptome of the prefrontal cortex of cisplatin-treated mice versus PBS-treated control mice. The top upregulated gene out of the 27 differentially expressed genes as a result of cisplatin treatment was *Cdh1*, the gene encoding the adhesion molecule E-cadherin. E-cadherin is a 120 kD transmembrane glycoprotein that regulates adhesion [64]. It is a component of the adherens junctions that stabilize the architecture of the non-compact myelin region in the peripheral nervous system. Deletion of the *Cdh1* gene in vivo delays myelination of peripheral axons [3]. In the brain, E-cadherin also plays a role at mature synapses where it regulates dynamic aspects of synaptic signaling, structural plasticity, and cognitive function [21]. Expression of two other genes involved in myelination was altered in the PFC of cisplatin-treated mice: *Mal* and *Ptgds (*prostaglandin D2 synthase). Prostaglandin D2 synthase is expressed by oligodendrocytes and is increased in models of demyelination [4, 56]. *Mal* encodes a proteolipid (MAL) produced by oligodendrocytes that is mainly localized in compact myelin where it colocalized with PLP and MBP [20, 47, 48]. MAL is thought to play a key role in stabilization of myelin membrane domains and in the maintenance of axon–glia interactions [19]. Cisplatin-induced changes in the expression of these genes may well contribute to the observed decompaction of myelin, but their exact role in this model remains to be determined.

In our search to reverse the adverse effects of cisplatin on myelination and the associated impairments at the level of cognitive and sensorimotor function, we explored the RXR agonist bexarotene. Bexarotene is an efficacious stimulator of the RXR and a synthetic product structurally similar to retinoic acid compounds [6]. It has a high central nervous system penetrance and is approved by the FDA for treatment of cutaneous T cell lymphoma [17]. We show here for the first time that only a 5 days treatment course with bexarotene is sufficient to restore both cognitive deficits and sensorimotor deficits in mice treated with cisplatin. We also show that a short 5 day treatment with bexarotene was capable of reversing the microscopic and ultrastructural changes in myelin that occurred as a result of cisplatin treatment. An initial study on the potential beneficial effect of bexarotene in mouse models of Alzheimer disease reported improved cognitive function accompanied by rapid clearance of beta-amyloid, but these findings were not replicated in later studies [2, 11, 42, 59]. In a model of traumatic brain injury, treatment with bexarotene improved sensorimotor and cognitive function [23]. In this traumatic brain injury model, the beneficial effects of bexarotene were associated with a reduction in proinflammatory cytokine production and microglia and astrocyte activation. We published previously that we did not detect signs of immune activation in the brain of cisplatin treated mice [7, 8]. In those studies, we used RNA seq analysis of the hippocampus, RT-PCR analysis of expression of cytokines in cortex and hippocampus, and immunofluorescence analysis of signs of microglia or astrocyte activity in multiple brain regions [7, 8]. Consistent with these previous findings, our current RNA seq analysis of the sensorimotor cortex did not detect signs of immune activation in the cisplatin-treated mice (Table 1). In addition, we did not detect major changes in inflammatory pathways in response to bexarotene treatment (Fig. 7). Therefore it is unlikely that inhibition of inflammation is the primary mechanism underlying the beneficial effects of bexarotene on myelin (ultra)structure and on cognitive and sensorimotor function in cisplatin-treated mice.

Zhou et al. recently showed that activation of the PPARβ/RXRa complex is essential for mature myelin maintenance [66]. They showed that depletion of the *Qki* gene, a co-activator of PPARβ/RXR caused demyelination in the adult mouse brain that was associated with a downregulation of the myelin lipids without changes in the protein content of myelin. Treatment of oligodendrocyte-specific *Qki* knockout mice with bexarotene normalized myelin lipid composition as assessed in the spinal cord, promoted resolution of the neurological disability and prolonged survival of the *Qki* knockout mice. Based on that study, we used lipidomic analysis to investigate whether the cisplatin-induced change in myelin structure and density and/or myelin normalization in response to bexarotene were associated with a change in lipid composition. However, cisplatin did not cause major changes in lipid composition when assessed 5 days after completion of treatment and we did not observe increases in brain lipids or changes in their composition in response to 5-day bexarotene treatment. These findings indicate that the beneficial effect of bexarotene on the abnormalities in myelin (ultra)structure in the brain of cisplatin-treated mice are not mediated via overall activation of lipid biosynthesis.

Consistent with its proposed activity as an RXR agonist, RNA sequencing analysis of the prefrontal cortex clearly showed that administration of bexarotene to cisplatin-treated mice induced activation of an RXR-heterodimer pathway; the TR/RXR-pathway was identified as one of the top canonical pathways. In addition, the LXR/RXR and PPAR/RXR pathways were activated in bexarotene-treated mice. It remains to be determined how activation of these RXR pathways could promote restoration of myelin and improve cognitive and sensorimotor function.

Pathway enrichment analysis of our RNA sequencing data showed activation of the Neuregulin signaling pathway and the Netrin signaling pathway (see Additional file 1: Supplementary Figures 3 and 4 for details) in the prefrontal cortex of mice treated with cisplatin followed by bexarotene compared to mice treated with cisplatin alone. The neuregulin pathway has been shown to be involved in myelination and synaptic function [34]. Neuregulins are a four member family of epidermal growth factor-like signaling molecules that have trophic function. In addition, neuregulins can switch oligodendrocytes from an activity independent mode into an activity dependent mode of myelination by increasing NMDA receptor currents in cell of the oligodendrocyte lineage [34]. Neuregulins activate AKT signaling, and we detected increased *Akt* expression in response to bexarotene (Additional file 1: Supplementary Figure 3). AKT activation promotes survival of oligodendrocytes in models of brain injury [18]. AKT signaling is also an important stimulus for mitochondrial function. We have shown previously that cisplatin treatment leads to mitochondrial dysfunction in brain synaptosomes [7, 8, 35]. It might be that neuregulin-AKT signaling as a result of bexarotene contributes to restoration of mitochondrial function in neurons and myelinated axons. In line with this model, triggering the PPAR/RXR with bexarotene restored impaired oxidative respiration in neurons thereby normalizing their bioenergetics status [13]. Together normalization of the bioenergetic status of neurons and oligodendrocytes could underlie the restoration of myelin integrity.

Netrin signaling is involved in many brain functions, and its role in myelination during development is well established [61]. In the adult brain, netrin signaling has dual effects on myelination. In demyelinated lesions in models of MS, netrin limits recruitment of oligodendrocyte precursor cells while it promotes differentiation into mature oligodendrocytes [57, 61]. In a model of ischemic stroke, overexpression of netrin-1 promoted long-time recovery by improving oligodendrogenesis and repair of white matter damage [24]. In our data set, netrin-1 was one of the genes in the netrin pathway that was upregulated in response to bexarotene administration to cisplatin treated mice (Additional file 1: Supplementary Figure 4).

## Conclusions

We show that only a short 5 day course of bexarotene, starting 1 day after completion of cisplatin treatment, is enough to reverse the cognitive dysfunction and structural deficits in the brain, implying a disease-modifying effect. This beneficial effect of a short course of bexarotene treatment is important because there is evidence from a clinical trial that 4 weeks of treatment with bexarotene can lead to high triglyceride levels presenting a danger for cardiovascular complications which decreased the enthusiasm to use the drug for an extended period of time. We propose that a short intervention with bexarotene after termination of cisplatin treatment is unlikely to have a negative effect of triglyceride levels. Future studies should examine whether the capacity of bexarotene to reverse cisplatin-induced cognitive deficits is retained in tumor bearing mice. If so, our findings could be rapidly developed into a treatment to resolve or prevent the devastating effects on quality of life of cancer survivors.

## Supplementary information

**Additional file 1:** **Supplementary figure 1.** RNAseq analysis of the effect of cisplatin on the transcriptome in the PFC. The heat map shows the differentially expressed genes from the comparison of the transcriptome of mice treated with PBS or cisplatin. **Supplementary Figure 2.** Effect of cisplatin and bexarotene on performance in the puzzle box test. The puzzle box test was performed 7 days after the last dose of Bexarotene treatment. The test measures time to escape from a brightly lit to a dark compartment connected by a tunnel. It consists of 3 levels of difficulty-easy (open tunnel; trials 1-4), intermediate (tunnel filled with bedding; trials 5-7), and difficult (tunnel covered with plug; trials 8-11). Results are expressed as mean ± SEM. A; Males, *n*=8; B: females, *n* = 8. Tukey’s post hoc **p <0.01 compared to PBS controls. **Supplementary Figure 3.** Neuregulin Pathway enrichment in cisplatin and bexarotene treated samples. Neuregulin Signaling pathway as identified by IPA analysis of differentially expressed genes in response to administration of bexarotene to cisplatin-treated mice. The up and down-regulated genes are shown in red and green respectively. **Supplementary Figure 4.** Netrin Pathway enrichment in cisplatin and bexarotene treated samples. Netrin Signaling pathway as identified by IPA analysis of differentially expressed genes in response to administration of bexarotene to cisplatin-treated mice. The up and downregulated genes are shown in red and green respectively.

**Additional file 2:** Differentially expressed genes padj<0.1 comparing cisplatin and cisplatin followed by bexarotene groups.

## Data Availability

The datasets used and/or analysed during the current study are  available from the corresponding author on reasonable request.
