# Supplementary material for: Bexarotene normalizes chemotherapy-induced myelin decompaction and reverses cognitive and sensorimotor deficits in mice
Source: Acta Neuropathol Commun. 2020 Nov 12;8:193. doi: 10.1186/s40478-020-01061-x (PMC7661216; doi:10.1186/s40478-020-01061-x)
Supplement: Supplementary file 1 — Additional file 1: Supplementary figure 1. RNAseq analysis of the effect of cisplatin on the transcriptome in the PFC. The heat map shows the differentially expressed genes from the comparison of the transcriptome of mice treated with PBS or cisplatin. Supplementary Figure 2. Effect of cisplatin and bexarotene on performance in the puzzle box test. The puzzle box test was performed 7 days after the last dose of Bexarotene treatment. The test measures time to escape from a brightly lit to a dark compartment connected by a tunnel. It consists of 3 levels of difficulty-easy (open tunnel; trials 1-4), intermediate (tunnel filled with bedding; trials 5-7), and difficult (tunnel covered with plug; trials 8-11). Results are expressed as mean ± SEM. A; Males, n=8; B: females, n = 8. Tukey’s post hoc **p <0.01 compared to PBS controls. Supplementary Figure 3. Neuregulin Pathway enrichment in cisplatin and bexarotene treated samples. Neuregulin Signaling pathway as identified by IPA analysis of differentially expressed genes in response to administration of bexarotene to cisplatin-treated mice. The up and down-regulated genes are shown in red and green respectively. Supplementary Figure 4. Netrin Pathway enrichment in cisplatin and bexarotene treated samples. Netrin Signaling pathway as identified by IPA analysis of differentially expressed genes in response to administration of bexarotene to cisplatin-treated mice. The up and downregulated genes are shown in red and green respectively. [file 40478_2020_1061_MOESM1_ESM.docx]

**
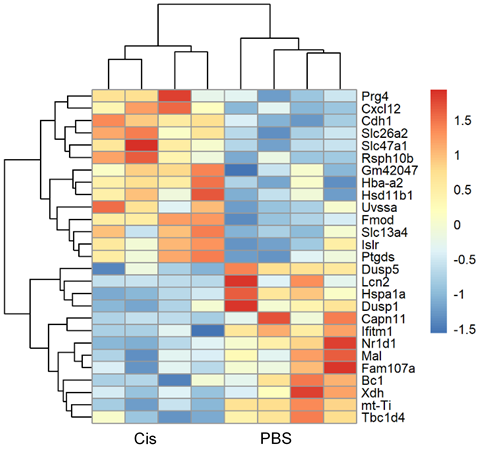
**

**Supplementary figure 1: RNAseq analysis of the effect of cisplatin on the transcriptome in the PFC**

The heat map shows the differentially expressed genes from the comparison of the transcriptome of mice treated with PBS or cisplatin

**Supplementary Figure 2. Effect of cisplatin and bexarotene on performance in the puzzle box test.**

The puzzle box test was performed 7 days after the last dose of Bexarotene treatment. The test measures time to escape from a brightly lit to a dark compartment connected by a tunnel. It consists of 3 levels of difficulty- easy (open tunnel; trials 1-4), intermediate (tunnel filled with bedding; trials 5-7), and difficult (tunnel covered with plug; trials 8-11). Results are expressed as mean ± SEM. A; Males, n=8; B: females, n = 8. Tukey’s post hoc **p <0.01 compared to PBS controls.

**Supplementary Figure 3: Neuregulin Pathway enrichment in cisplatin and bexarotene treated samples.
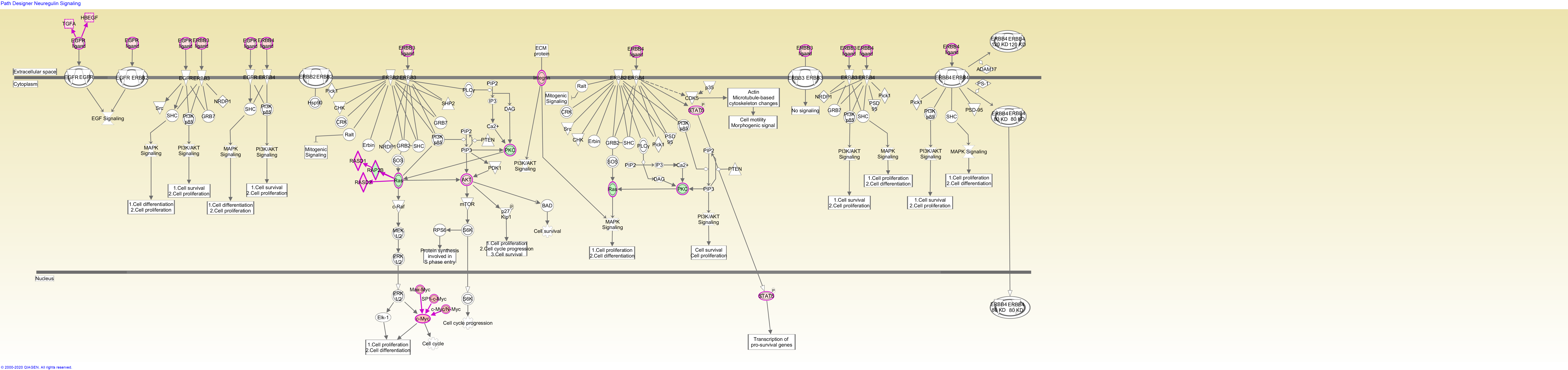
**Neuregulin Signaling pathway as identified by IPA analysis of differentially expressed genes in response to administration of bexarotene to cisplatin-treated mice. The up and down-regulated genes are shown in red and green respectively.

**
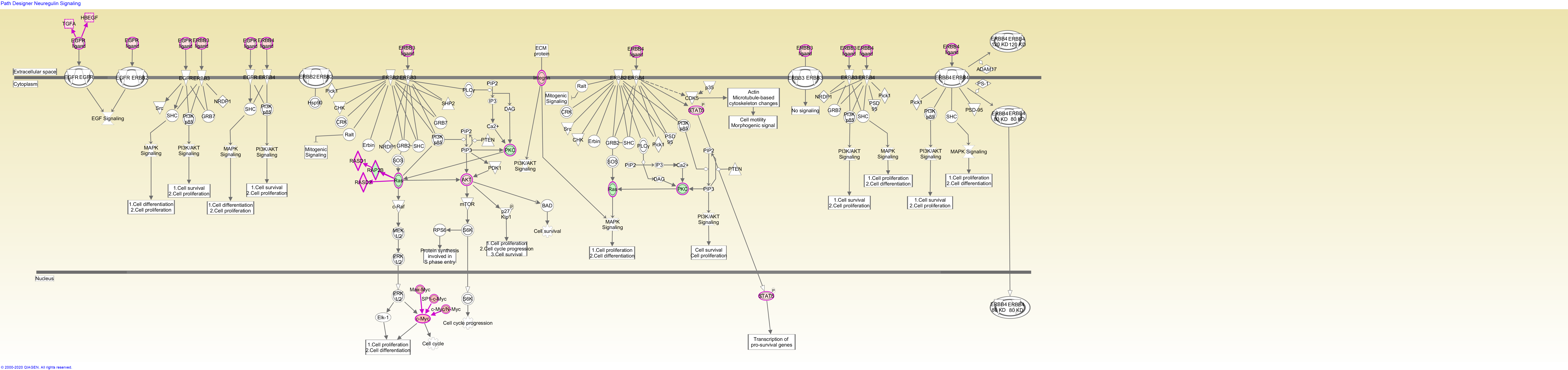
**

**
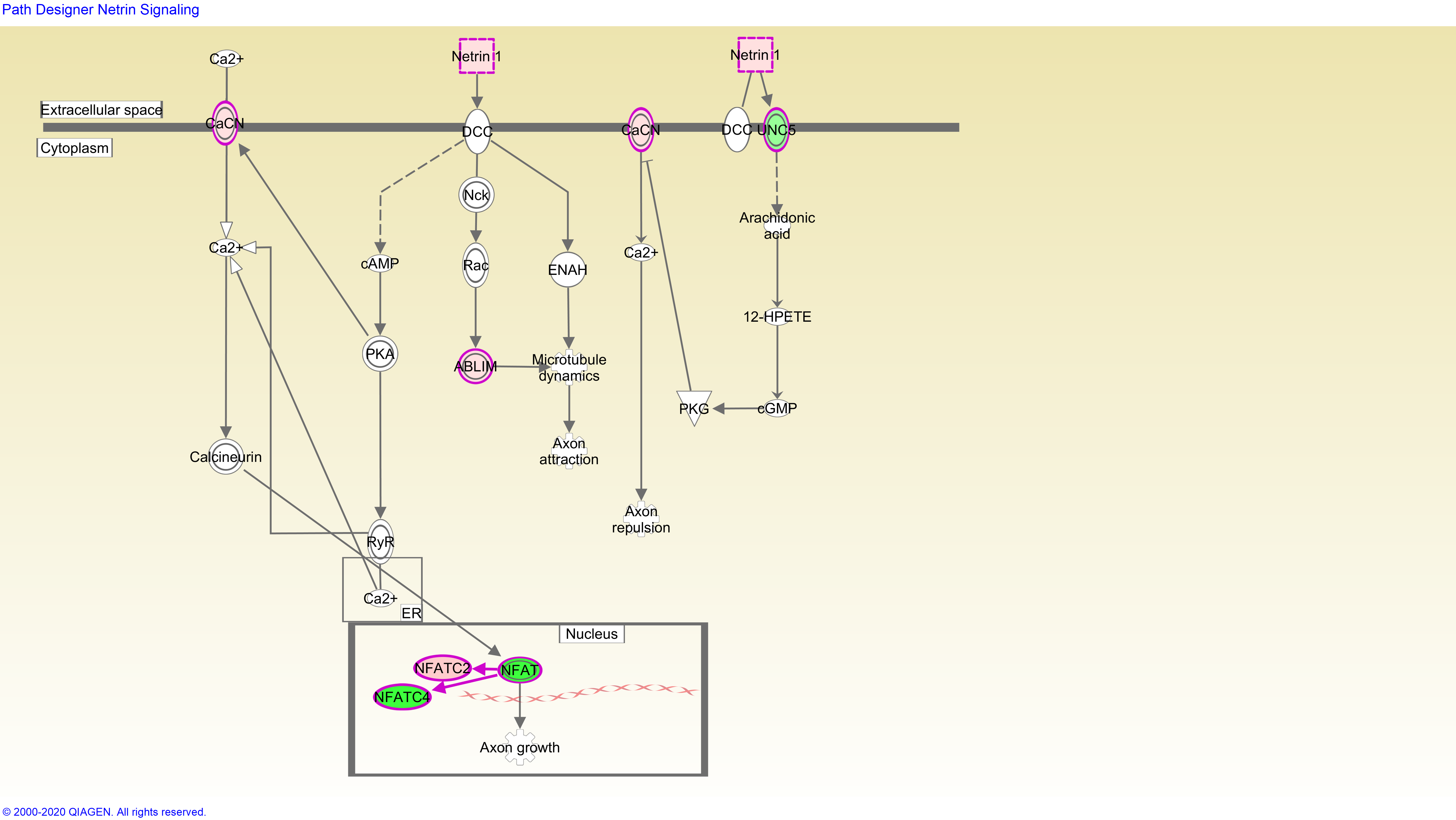
Supplementary Figure 4: Netrin Pathway enrichment in cisplatin and bexarotene treated samples.** Netrin Signaling pathway as identified by IPA analysis of differentially expressed genes in response to administration of bexarotene to cisplatin-treated mice. The up and down-regulated genes are shown in red and green respectively.
